# Prognostic Effect of Neck Dissection and Risk Factors for Occult Lymph Node Metastasis in cN0 Parotid Carcinoma

**DOI:** 10.1002/cai2.70007

**Published:** 2025-04-07

**Authors:** Yudong Ning, Yixuan Song, Yuqin He, Han Li, Shaoyan Liu

**Affiliations:** ^1^ Department of Head and Neck Surgery, National Cancer Center/National Clinical Research Center for Cancer/Cancer Hospital Chinese Academy of Medical Sciences and Peking Union Medical College Beijing China

**Keywords:** clinically negative lymph node, cN0, neck dissection, occult lymph node metastasis, parotid carcinoma

## Abstract

**Objective:**

This study aimed to explore the prognostic effect of neck dissection and to identify risk factors associated with occult lymph node metastasis (OLNM) in clinically node‐negative (cN0) parotid carcinoma (PC).

**Methods:**

A retrospective analysis was conducted on cN0 PC patients who underwent primary surgery at the National Cancer Center/Cancer Hospital, Chinese Academy of Medical Sciences, between 2012 and 2022. Kaplan–Meier (KM) survival analyses were carried out to evaluate differences in progression‐free survival (PFS) and overall survival (OS) between patients undergoing neck dissection and those who did not. Clinical variables associated with OLNM in the neck dissection group were assessed using univariate and multivariate logistic regression analyses.

**Results:**

Among 472 PC patients, 133 were classified as cN0 following initial surgery, of whom 75 (56.4%) underwent neck dissection. Pathological lymph node metastases were confirmed in 20 (26.7%) patients in the neck dissection cohort. Poor tumor differentiation was identified as an independent risk factor for OLNM (*p* = 0.017). No significant differences in PFS or OS were observed between the no‐neck dissection and neck dissection groups for patients with low‐grade or well‐differentiated tumors (*p* > 0.05). However, neck dissection was associated with significantly prolonged PFS in patients with tumors of higher grade or low to moderate differentiation (*p* < 0.05). Notably, OS did not improve with neck dissection across all subgroups (*p* > 0.05).

**Conclusion:**

Poorly differentiated tumors in cN0 PC are independently associated with a higher risk of OLNM. While prophylactic neck dissection may enhance PFS in patients with higher grade or poorly differentiated tumors, it does not confer a survival benefit in terms of OS. These findings support the selective use of neck dissection in patients with higher risk tumor profiles.

## Graphical abstract

For cN0 parotid carcinoma, preventive neck dissection may prolong PFS of higher grade tumor types or low‐ and moderate‐differentiation PC, although it does not improve OS. Therefore, neck dissection should be performed in these cohorts.

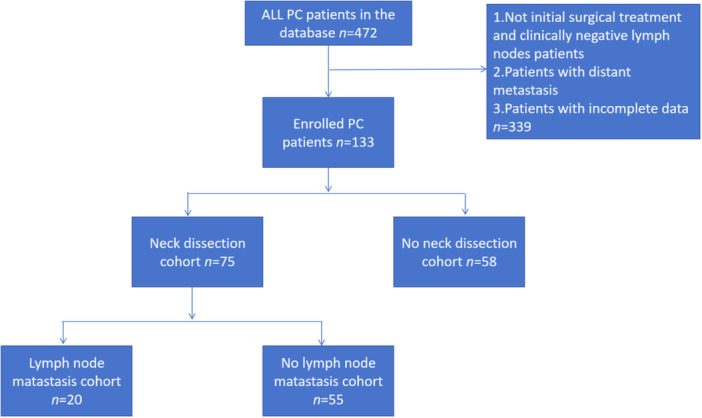

**Abbreviations**
AJCC: American Joint Committee on Cancer
cN0: clinically node‐negative
cN^+^
: clinically node‐positive
CT: computed tomography
KM: Kaplan–Meier
MRI: magnetic resonance imaging
OLNM: occult lymph node metastasis
OS: overall survival
PC: parotid carcinoma
PFS: progression‐free survival
WHO: World Health Organization

## 1 Introduction

Salivary gland carcinomas constitute approximately 3% of all head and neck malignancies [1]. These neoplasms primarily arise in the major salivary glands, including the parotid, submandibular, and sublingual glands, as well as in the minor salivary glands distributed throughout the oral mucosa. Among salivary gland tumors, parotid gland tumors represent 80% of cases, with 20%–25% of these being malignant [2, 3]. Surgical resection remains the cornerstone of treatment for primary parotid carcinoma (PC), and neck dissection is the recommended approach for patients presenting with clinically node‐positive (cN^+^) disease [4]. However, the role of neck dissection in clinically node‐negative (cN0) patients remains contentious [5, 6]. Current management strategies for cN0 PC patients vary and include observation, prophylactic neck dissection, and elective radiotherapy [7, 8, 9, 10]. The reported prevalence of occult lymph node metastasis (OLNM) in cN0 PC patients has a wide range, from 0% to 60% [11, 12, 13, 14, 15]. The primary aim of neck dissection in cN0 PC patients is to excise lymph nodes harboring OLNM [5, 16]. Despite its utility, neck dissection is an invasive procedure that can result in additional surgical morbidity, raising concerns about its routine application in all cN0 cases. Retrospective analyses have identified various prognostic factors associated with an increased risk of OLNM, suggesting that select patients may derive benefit from this intervention in the context of locoregional disease management [17, 18]. Nevertheless, robust clinical evidence regarding the survival benefits of neck dissection in cN0 PC remains scarce. The rarity of PC and its diverse histopathological subtypes further complicate the development of standardized surgical protocols, hindering the precise determination of optimal management strategies for cN0 patients [19]. Consequently, there is no consensus in the literature regarding the most appropriate treatment approach for this patient population.

In this study, we analyzed data from our institution to evaluate the prognostic impact of prophylactic neck dissection in cN0 PC patients. Furthermore, we sought to identify high‐risk factors associated with OLNM, with the goal of providing evidence‐based guidance to inform clinical decision‐making.

## 2 Methods

### 2.1 Characteristics of the Study Cohort

This retrospective study evaluated 133 patients diagnosed with cN0 PC who underwent primary surgical intervention at the National Cancer Center/National Clinical Research Center for Cancer/Cancer Hospital, Chinese Academy of Medical Sciences, between 2012 and 2022. Among these, 75 patients underwent neck dissection, with 20 cases histopathologically confirmed to have lymph node metastases. All neck dissections were confined to levels I–III. Comprehensive clinical data were collected, including patient demographics (gender and age), tumor characteristics (type and differentiation), facial nerve status (invasion and preservation), T staging, neck dissection outcomes (lymph node metastases), adjuvant radiotherapy, progression‐free survival (PFS), and overall survival (OS). Facial nerve invasion was defined as close adherence of the tumor to the facial nerve. In cases of microinvasion, tumor resection was performed with preservation of the facial nerve. However, extensive infiltration necessitated the removal of the involved nerve segments. Tumor staging was performed according to the criteria outlined in the eighth edition of the American Joint Committee on Cancer (AJCC). Tumor classification adhered to the 2022 (fifth edition) World Health Organization (WHO) criteria for salivary gland neoplasms, with tumors categorized into low‐grade and non‐low‐grade types based on histopathology and differentiation [20]. Low‐grade PC included highly differentiated mucoepidermoid carcinomas, adenoid cystic carcinomas, myoepithelial carcinomas, pleomorphic adenoma carcinomas, acinar cell carcinomas, and basal cell carcinomas. Conversely, non‐low‐grade PC included low‐differentiated and moderately differentiated mucoepidermoid carcinomas, adenoid cystic carcinomas, myoepithelial carcinomas, pleomorphic adenoma carcinomas and all squamous cell carcinomas, ductal carcinomas, adenocarcinomas, and lymphoepithelial carcinomas. PFS was defined as the duration from surgery to the first instance of tumor progression, including local recurrence or distant metastasis. OS was defined as the time from surgery to the final follow‐up or patient death. All clinical and pathological data were meticulously documented.

### 2.2 Inclusion and Exclusion Criteria

The inclusion criteria for this study comprised the following: (1) all patients with cN0 PC who underwent initial and surgical treatment; (2) absence of distant metastasis; and (3) availability of complete clinical data. Patients were excluded if they met any of the following criteria: (1) presence of cN^+^ PC; (2) recurrent PC; (3) distant metastasis; or (4) incomplete clinical data.

### 2.3 Diagnostic Evaluation and Treatment Method

All patients included in the study underwent preoperative diagnostic imaging, which consisted of color Doppler ultrasound, contrast‐enhanced computed tomography (CT), or contrast‐enhanced magnetic resonance imaging (MRI).

Tumor classification was established through cytological analysis obtained via ultrasound‐guided fine‐needle aspiration based on the Milan System for Reporting Salivary Gland Cytopathology or intraoperative pathological examination. Surgical intervention involved resection of the parotid gland and tumor, with or without concurrent neck dissection. Postoperative radiotherapy was administered as indicated.

### 2.4 Statistical Analysis

Baseline characteristics of patients in the cohorts undergoing neck dissection and those without neck dissection were compared using the chi‐square test. PFS and OS were analyzed and visualized using Kaplan–Meier (KM) curves, stratified by tumor type and differentiation. Risk factors for OLNM in the neck dissection cohort were assessed using both univariate and multivariate binary logistic regression analyses. Statistical significance was defined as *p* < 0.05.

## 3 Results

### 3.1 Cohort Characteristics

**Figure 1:**
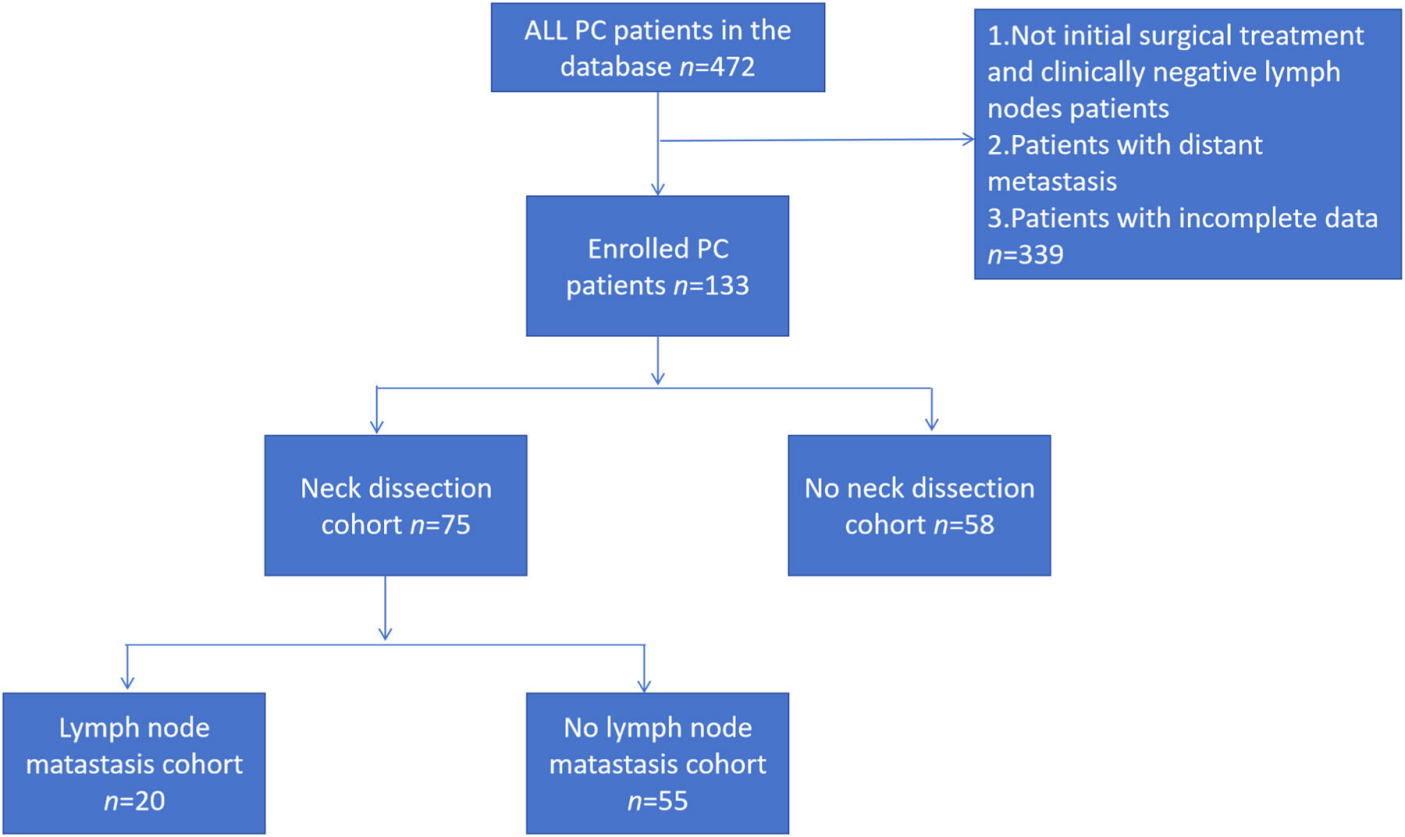
Flow diagram of the present study. PC, parotid carcinoma.

The cohort comprised 472 patients, among whom 133 were diagnosed with cN0 PC following initial surgical intervention (Figure 1, Table 1). Of these patients, 75 (56.4%) underwent neck dissection, and pathological examination confirmed lymph node metastases in 20 cases (26.7%). Patients were stratified by gender and further categorized based on tumor type into two groups: low‐grade malignancies (71 patients, 53.4%) and non‐low‐grade malignancies (62 patients, 46.6%). Tumor differentiation status was distributed as follows: high differentiation (82 patients, 61.7%), moderate differentiation (26 patients, 19.5%), and low differentiation (25 patients, 18.8%). Additionally, 56 patients (42.1%) showed facial nerve invasion. According to the eighth edition of the TNM staging system, patients were classified as follows: T1 (54 patients, 40.6%), T2 (69 patients, 51.9%), T3 (8 patients, 6.0%), and T4 (2 patients, 1.5%). Facial nerve resection status was documented, with 119 patients (89.5%) undergoing facial nerve‐sparing surgery, while 14 patients (10.5%) underwent resection.

**Table 1:** Characteristics of the cohort.

Items	Number (%), *N* = 133
Gender	
Female	72 (54.1)
Male	61 (45.9)
Age (years old)	
< 60	94 (70.7)
≥ 60	39 (29.3)
Tumor types (low‐grade malignancy)	
No	62 (46.6)
Yes	71 (53.4)
Differentiation	
High	82 (61.7)
Mid	26 (19.5)
Low	25 (18.8)
Facial nerve invaded	
No	77 (52.9)
Yes	56 (42.1)
Facial nerve reserved	
No	14 (10.5)
Yes	119 (89.5)
T Staging	
T1	54 (40.6)
T2	69 (51.9)
T3	8 (6.0)
T4	2 (1.5)
Neck dissection	
No	58 (43.6)
Yes	75 (56.4)

As shown in Table 1, neck dissection was performed in 75 patients (56.4%), among whom 20 (26.7%) were confirmed to have lymph node metastases through pathological examination. The clinical incidence rates of OLNM are detailed in Table 2. These rates were stratified by demographic and clinical factors: females (7, 18.4%), males (13, 35.1%), patients aged ≥ 60 years (8, 32.0%), patients aged < 60 years (12, 24.0%), low‐grade tumor types (6, 17.7%), non‐low‐grade tumor types (14, 34.2%), high differentiation (6, 16.2%), moderate differentiation (4, 19.0%), low differentiation (10, 58.8%), facial nerve invasion (13, 37.1%), no facial nerve invasion (7, 17.5%), preservation of the facial nerve (16, 24.2%), non‐preservation of the facial nerve (4, 44.4%), T1–T2 stages (19, 27.9%), and T3–T4 stages (1, 14.3%).

**Table 2:** Characteristics, and univariate and multivariate analyses of lymph node metastasis based on neck dissection.

Clinical characters	Neck dissection number, *N* = 75, *n* (%)	Lymph node metastasis, *N* = 20, *n* (%)	Univariate OR (95% CI)	*p* value	Multivariate OR (95% CI)	*p* value
Gender						
Female	38 (54.1)	7 (18.4)				
Male	37 (45.9)	13 (35.1)	0.875 (0.829–6.939)	0.106	1.184 (0.95–11.23)	0.16
Age (years old)						
< 60	50 (70.7)	12 (24.0)				
≥ 60	25 (29.3)	8 (32.0)	0.399 (0.515–4.309)	0.462	1.096 (0.727–12.31)	0.129
Tumor types (low‐grade malignancy)						
No	34 (53.4)	14 (34.2)	0.884 (0.811–7.21)	0.113	2.138 (0.008–1.77)	0.122
Yes	41 (46.6)	6 (17.7)				
Differentiation						
High	37 (61.7)	6 (16.2)				
Moderate	21 (19.5)	4 (19.0)				
Low	17 (18.8)	10 (58.8)	0.987 (1.371–5.252)	0.004	1.883 (1.392–31.05)	0.017
Facial nerve invaded						
No	40 (57.9)	7 (17.5)				
Yes	35 (42.1)	13 (37.1)	1.025 (0.960–8.085)	0.059	1.201 (0.766–14.43)	0.109
Facial nerve reserved						
No	9 (10.5)	4 (44.4)				
Yes	66 (89.5)	16 (24.2)	−0.916 (0.96–1.672)	0.209	−0.104 (0.156–5.19)	0.907
T Staging						
T1–T2	68 (92.5)	19 (27.9)				
T3–T4	7 (7.5)	1 (14.3)	0.844 (0.048–3.811)	0.448	1.443 (0.019–2.94)	0.262

Abbreviations: CI, confidence interval; OR, odd ratio.

Low‐grade PC was categorized as including highly differentiated mucoepidermoid carcinoma, highly differentiated adenoid cystic carcinoma, highly differentiated myoepithelial carcinoma, highly differentiated pleomorphic adenoma carcinomas, acinar cell carcinoma, and basal cell carcinoma. In contrast, non‐low‐grade PC encompassed low‐differentiated and moderately differentiated mucoepidermoid carcinomas, low‐differentiated and moderately differentiated adenoid cystic carcinomas, low‐differentiated and moderately differentiated myoepithelial carcinomas, low‐differentiated and moderately differentiated pleomorphic adenoma carcinomas, and all cases of squamous cell carcinoma, ductal carcinoma, adenocarcinoma, and lymphoepithelial carcinoma. The OLNM rates associated with specific pathological tumor subtypes are presented in Tables 3 and 4. Notably, the OLNM rates were substantially higher among non‐low‐grade tumor types, including ductal carcinoma (80%), adenocarcinoma (75%), lymphoepithelial carcinoma (50%), and squamous cell carcinoma (30%), compared with low‐grade tumor types.

**Table 3:** The characteristics and lymph node metastasis of pathological types.

Pathological types	Number *N* = 75, *n* (%)	Lymph node metastasis *N* = 20, *n* (%)
**Mucoepidermoid carcinoma**	**30 (40.0)**	**4 (13.3)**
High differentiation	13	1
Middle differentiation	13	1
Low differentiation	4	2
**Adenoid cystic carcinoma**	**9 (12.0)**	**3 (33.3)**
High differentiation	7	3
Low differentiation	2	0
**Myoepithelial carcinoma**	**1 (1.3)**	**0 (0.0)**
High differentiation	1	0
**Malignant pleomorphic adenoma**	**5 (6.7)**	**0 (0.0)**
High differentiation	4	0
Low differentiation	1	0
**Acinic cell carcinoma**	**9 (12.0)**	**2 (22.2)**
High differentiation	9	2
**Ductal carcinoma**	**5 (6.7)**	**4 (80.0)**
High differentiation	1	0
Low differentiation	4	4
**Adenocarcinoma**	**4 (5.3)**	**3 (75.0)**
High differentiation	1	0
Low differentiation	3	3
**Lymph epithelial carcinomas**	**2 (2.7)**	**1 (50.0)**
High differentiation	1	0
Low differentiation	1	1
**Squamous cell carcinoma**	**10 (13.3)**	**3 (30.0)**
Middle differentiation	8	3
Low differentiation	2	0

**Table 4:** The characteristics and lymph node metastasis of tumor types.

Tumor types	Number, *N* = 75, *n* (%)	Lymph node metastasis, *N* = 20, *n* (%)
**Low grades**	**34 (45.3)**	**6 (17.7)**
**Mucoepidermoid carcinoma**		
High differentiation	13 (17.3)	1 (7.70)
**Adenoid cystic carcinoma**		
High differentiation	7 (9.3)	3 (42.9)
**Myoepithelial carcinoma**		
High differentiation	1 (1.3)	0 (0.0)
**Malignant pleomorphic adenoma**		
High differentiation	4 (5.3)	0 (0.0)
**Acinic cell carcinoma**		
High differentiation	9 (12.0)	2 (22.2)
**Non low grades**	41 (54.7)	14 (34.2)
**Mucoepidermoid carcinoma**		
Middle differentiation	13 (17.3)	1 (7.70)
Low differentiation	4 (5.3)	2 (50.0)
**Adenoid cystic carcinoma**		
Low differentiation	2 (2.7)	0 (0.0)
**Malignant pleomorphic adenoma**		
Low differentiation	1 (1.3)	0 (0.0)
**Ductal carcinoma**		
High differentiation	1 (1.3)	0 (0.0)
Low differentiation	4 (5.3)	4 (100)
**Adenocarcinoma**		
High differentiation	1 (1.3)	0 (0.0)
Low differentiation	3 (4.0)	3 (100)
**Lymph epithelial carcinomas**		
High differentiation	1 (1.3)	0 (0.0)
Low differentiation	1 (1.3)	1 (100)
**Squamous cell carcinoma**		
Middle differentiation	8 (10.7)	3 (37.5)
Low differentiation	2 (2.7)	0 (0.0)

### 3.2 Analysis of Risk Factors for OLNM in cN0 PC

The analysis of risk factors for OLNM in cN0 PC was conducted using univariate and multivariate binary logistic regression models, with the results summarized in Table 5. Univariate analysis identified poor tumor differentiation to be significantly associated with OLNM (OR: 0.987, 95% CI: 1.371–5.252, *p* = 0.004). Further multivariate analysis confirmed poor differentiation as an independent risk factor for OLNM (OR: 1.883, 95% CI: 1.392–31.05, *p* = 0.017). The rates of OLNM stratified by differentiation grade were as follows: highly differentiated tumors (6, 16.2%), moderately differentiated tumors (4, 19.0%), and poorly differentiated tumors (10, 58.8%).

**Table 5:** Baseline characteristics between no neck dissection and neck dissection in high differentiation.

Clinical characteristics	No neck dissection, *N* = 45, *n* (%)	Neck dissection, *N* = 37, *n* (%)	*p* value
Gender			
Female	28 (62.2)	19 (51.4)	0.318
Male	17 (37.8)	18 (48.6)	
Age (years old)			
< 60	35 (77.8)	26 (70.3)	0.538
≥ 60	10 (22.2)	11 (29.7)	
Facial nerve invaded			
No	29 (64.4)	23 (62.2)	0.831
Yes	16 (35.6)	14 (37.8)	
Facial nerve reserved			
No	4 (8.9)	4 (10.8)	0.770
Yes	41 (91.1)	33 (89.2)	
T State			
T1–T2	43 (95.6)	33 (89.2)	0.094
T3–T4	2 (4.4)	4 (10.8)	

### 3.3 Prognostic Effect of Neck Dissection Across Tumor Grades

**Figure 2:**
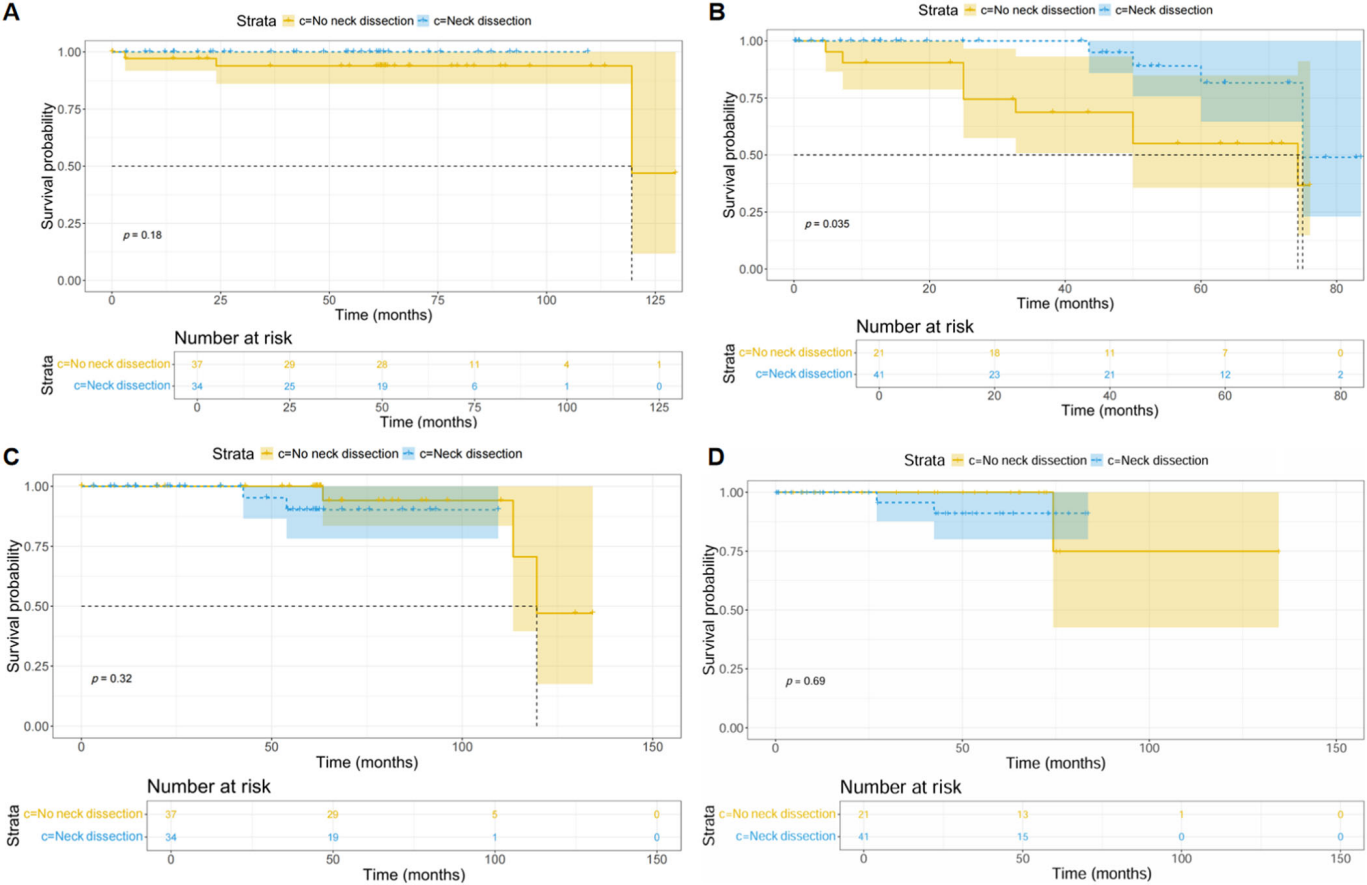
KM curve of low‐grade tumor types and non‐low‐grade tumor types. (A) PFS for low‐grade tumor types; (B) PFS for non‐low‐grade tumor types; (C) OS for low‐grade tumor types; and (D) OS for non‐low‐grade tumor types. (A) There were no significant differences between the no‐neck dissection cohort and the neck dissection cohort on PFS for low‐grade tumor types (*p* = 0.18). (B) The neck dissection cohort had a better PFS than the no dissection cohort for non‐low‐grade tumor types (*p* = 0.035). (C, D) There were no significant differences between the no‐neck dissection cohort and the neck dissection cohort on OS for low‐grade tumor types (*p* = 0.32) and non‐low‐grade tumor types (*p* = 0.69). KM, Kaplan–Meier; OS, overall survival; PFS, progression‐free survival.

The prognostic value of neck dissection was further evaluated for low‐grade and non‐low‐grade tumors based on clinical parameters. Baseline characteristics of the no‐neck dissection and neck dissection cohorts were compared using chi‐square tests, with the results presented in Tables 5 and 6. No statistically significant differences were observed between the two groups across baseline variables (*p* > 0.05; Tables 5 and 6). PFS analysis revealed no significant differences between the no‐neck dissection and neck dissection cohorts for low‐grade tumors (*p* = 0.18, Figure 2A). However, patients in the neck dissection cohort demonstrated significantly improved PFS compared with those in the no‐dissection cohort for non‐low‐grade tumor types (*p* = 0.035; Figure 2B). Analysis of OS indicated no significant differences between the two cohorts for both low‐grade (*p* = 0.32; Figure 2C) and non‐low‐grade tumor types (*p* = 0.69; Figure 2D).

**Table 6:** Baseline characteristics between no neck dissection and neck dissection in poor and moderate differentiation.

Clinical characters	No neck dissection, *N* = 13, *n* (%)	Neck dissection, *N* = 38, *n* (%)	*p* value
Gender			
Female	6 (46.2)	20 (52.6)	0.687
Male	7 (53.8)	18 (47.4)	
Age (years old)			
< 60	9 (69.2)	24 (63.2)	0.692
≥ 60	4 (30.8)	14 (36.8)	
Facial nerve invaded			
No	8 (61.5)	17 (44.7)	0.296
Yes	5 (38.5)	21 (55.3)	
Facial nerve reserved			
No	1 (7.7)	5 (13.2)	0.598
Yes	12 (92.3)	33 (86.8)	
T State			
T1–T2	12 (92.3)	35 (92.1)	0.676
T3–T4	1 (7.7)	3 (7.9)	

### 3.4 Prognosis Effect of Neck Dissection in Relation to Tumor Differentiation

**Figure 3:**
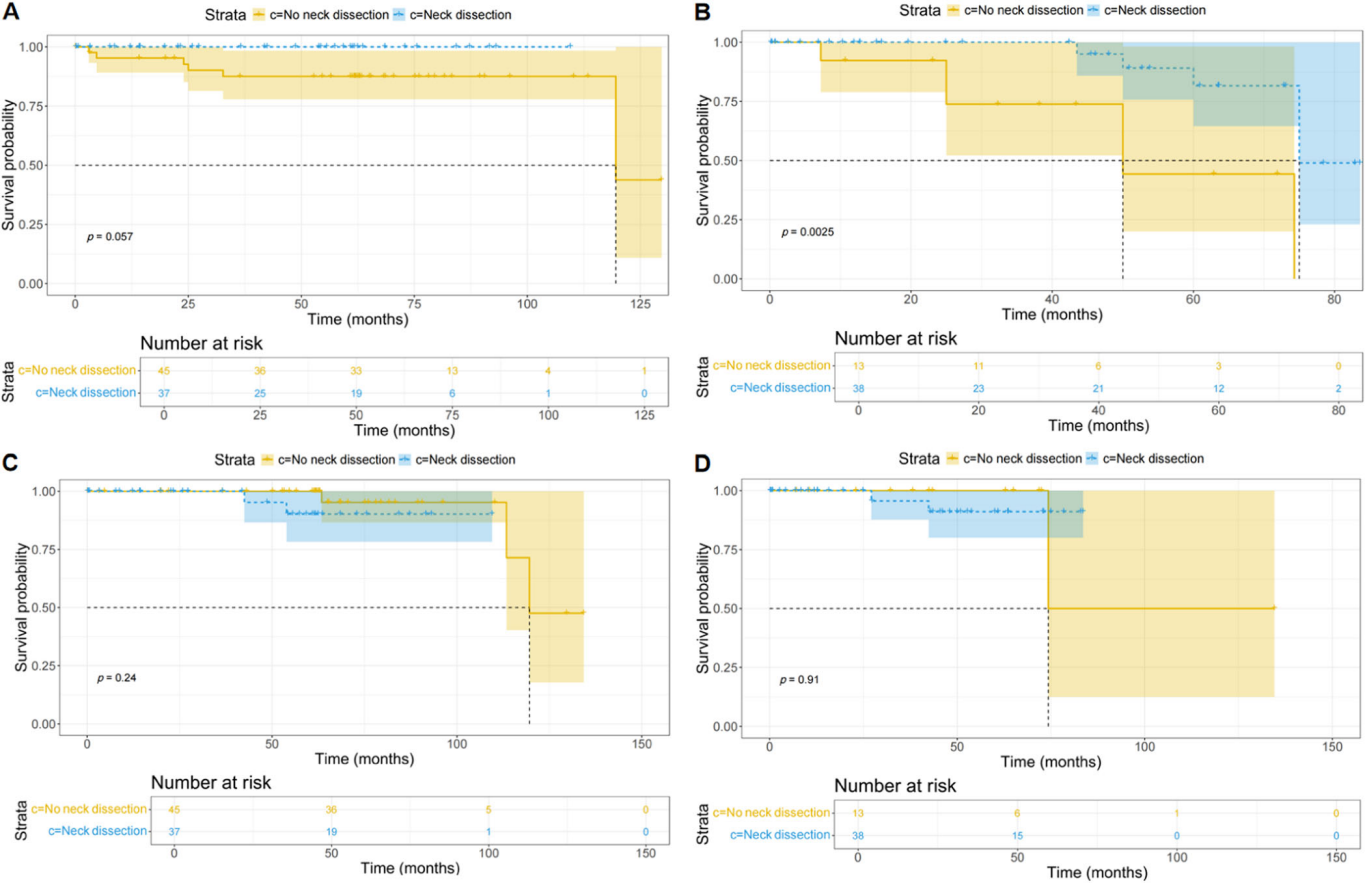
KM curve of high differentiation and low and moderate differentiation. (A) PFS for high differentiation; (B) PFS for low and moderate differentiation; (C) OS for high differentiation; and (D) OS for low and moderate differentiation. (A) There were no significant differences between the no‐neck dissection cohort and the neck dissection cohort on PFS for high differentiation (*p* = 0.057). (B) The neck dissection cohort had a better PFS than the no dissection cohort for low and moderate differentiation (*p* = 0.0025). (C, D) There were no significant differences between the no‐neck dissection cohort and the neck dissection cohort on OS for high differentiation (*p* = 0.24) and low and moderate differentiation (*p* = 0.91). KM, Kaplan–Meier; OS, overall survival; PFS, progression‐free survival.

Patients were categorized into two differentiation‐based groups: high differentiation and low‐to‐moderate differentiation. Baseline characteristics of the no‐neck and neck dissection cohorts were compared using chi‐square tests (Tables 7 and 8). No significant differences were identified between these cohorts across all baseline data (*p* > 0.05; Tables 7 and 8). For highly differentiated tumors, PFS did not differ significantly between the no‐neck and neck dissection cohorts (*p* = 0.057; Figure 3A). Conversely, for tumors with low‐to‐moderate differentiation, the neck dissection cohort showed significantly improved PFS compared to the no‐neck dissection cohort (*p* = 0.0025; Figure 3B). OS analysis revealed no significant differences between the no‐neck dissection and neck dissection cohorts for highly differentiated tumors (*p* = 0.24; Figure 3C) or for low‐to‐moderately differentiated tumors (*p* = 0.91; Figure 3D).

**Table 7:** Baseline characteristics between no neck dissection and neck dissection in low‐grade tumor types.

Clinical characteristics	No neck dissection, *N* = 37, *n* (%)	Neck dissection, *N* = 34, *n* (%)	*p* value
Gender			
Female	24 (64.9)	18 (52.9)	0.307
Male	13 (35.1)	16 (47.1)	
Age (years old)			
< 60	28 (75.7)	25 (73.5)	0.835
≥ 60	9 (24.3)	9 (26.5)	
Facial nerve invaded			
No	26 (70.3)	22 (64.7)	0.258
Yes	11 (29.7)	12 (35.3)	
Facial nerve reserved			
No	2 (5.4)	3 (8.8)	0.574
Yes	35 (94.6)	31 (91.2)	
T State			
T1–T2	35 (94.6)	32 (94.1)	0.597
T3–T4	2 (5.4)	2 (5.9)	

**Table 8:** Baseline characteristics between no neck dissection and neck dissection in non‐low‐grade tumor types.

Clinical characteristics	No neck dissection, *N* = 21, *n* (%)	Neck dissection, *N* = 41, *n* (%)	*p* value
Gender			
Female	10 (47.6)	20 (48.8)	0.931
Male	11 (52.4)	21 (51.2)	
Age (years old)			
< 60	16 (76.2)	25 (61.0)	0.231
≥ 60	5 (23.8)	16 (39.0)	
Facial nerve invaded			
No	11 (52.4)	18 (43.9)	0.527
Yes	10 (47.6)	23 (35.3)	
Facial nerve reserved			
No	3 (14.3)	6 (14.6)	0.971
Yes	18 (85.7)	35 (85.4)	
T State			
T1–T2	20 (95.2)	36 (87.8)	0.739
T3–T4	1 (4.8)	5 (12.2)	

### 3.5 The Prognosis Effect of Neck Dissection on Overall cN0 PC

**Figure 4:**
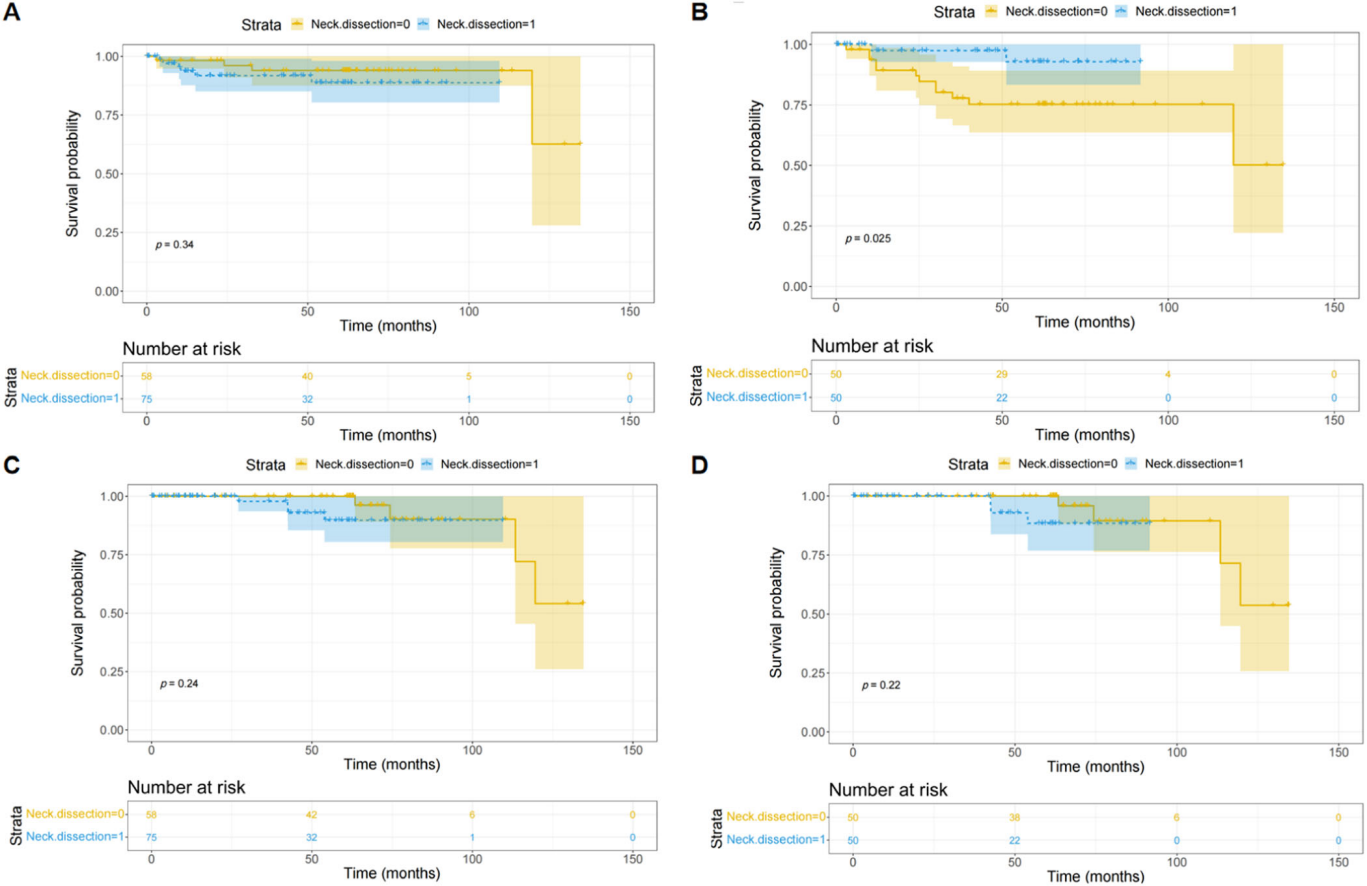
KM curve of neck dissection for all patients. (A) PFS before PSM; (B) PFS after PSM; (C) OS before PSM; and (D) OS after PSM. (A) There were no significant differences between the no‐neck dissection cohort and the neck dissection cohort on PFS before PSM (*p* = 0.34). (B) The neck dissection cohort had a better PFS than the no‐neck dissection cohort after PSM (*p* = 0.025). (C, D) There were no significant differences between the no‐neck dissection cohort and the neck dissection cohort on OS before and after PSM (*p* = 0.24, 0.22). KM, Kaplan–Meier; OS, overall survival; PFS, progression‐free survival; PSM, propensity score matching.

Supplementary Table 1 compares the baseline data of the no‐neck dissection cohort and the neck dissection cohort using a chi‐square test. The results showed significant differences in differentiation (*p* = 0.003), but no significant differences in other data (*p* > 0.05). After PSM, there were no significant differences between these two cohorts in all data (*p* > 0.05; Supplementary Table 2). There were no significant differences between the no‐neck dissection cohort and the neck dissection cohort on PFS before PSM (*p* = 0.34; Figure 4A). However, the neck dissection cohort had a better PFS than the no‐neck dissection cohort after PSM (*p* = 0.025; Figure 4B). Furthermore, there were no significant differences between the no‐neck dissection cohort and the neck dissection cohort on OS before and after PSM (*p* = 0.24, 0.22; Figure 4C,D).

## 4 Discussion

Cervical lymph node metastasis is a major adverse prognostic factor in patients with PC [21, 22]. However, the management of cN0 PC remains contentious, particularly regarding the routine application of neck dissection. While neck dissection is the standard therapeutic approach for cervical lymph node metastasis in PC, indiscriminate application may result in unnecessary surgical trauma. Therefore, precise identification of high‐risk patients is essential to guide prophylactic neck dissection.

Prophylactic neck dissection is recommended for individuals with identifiable high‐risk attributes. Studies have identified several factors associated with an increased risk of lymph node metastasis in PC, including high tumor grade, external parotid invasion, tumor size ≥ 4 cm, and facial nerve invasion [23]. Wang et al. [24] reported that nerve invasion, advanced T stage, and specific histological subtypes were primary determinants of lymph node metastasis in PC. Similarly, Stodulski et al. [25] observed a 30% incidence of OLNM in a cohort of 66 cN0 patients who underwent neck dissection. Univariate analysis revealed that intra‐parotid/periparotid metastasis, external parotid invasion, high T stage, and histology were significant risk factors for OLNM, while multivariate analysis identified intra‐parotid/periparotid metastasis as an independent predictor. Régis de Brito Santos et al. [23] reported OLNM in 37% (17 of 46) of cN0 patients who received neck dissection, with multivariate analysis identifying histological subtypes and T3–T4 staging as independent predictive factors. The UK National Multidisciplinary Guidelines recommend selective neck dissection for cN0 patients presenting with T3–T4 staging or high‐grade tumors, including squamous cell carcinoma, undifferentiated carcinoma, adenocarcinoma, high‐grade mucoepidermoid carcinoma, and pleomorphic adenoma carcinomas [2, 4]. Yoo et al. [26] further demonstrated that histologic grade was an independent and significant risk factor for lymph node metastasis. Armstrong et al. quantified the risk of OLNM in cN0 PC patients with T4 tumors at 24%, T3 tumors at 16%, and T1–T2 tumors at 7% [27]. Additionally, age has been identified as a potential predictor of lymph node metastasis in PC. Poulsen et al. [28] reported an increased risk among patients aged ≥ 60 years, while Klussmann et al. [29] similarly identified age ≥ 61 years as a significant risk factor.

The present data (Table 1) indicate that the overall rate of OLNM was 26.7%. Findings from univariate and multivariate binary logistic regression analyses of lymph node metastasis, based on neck dissection outcomes, are presented in Table 5. Univariate analysis identified poor differentiation to be significantly associated with OLNM in patients with cN0 PC (OR: 0.987, 95% CI: 1.371–5.252, *p* = 0.004). Multivariate analysis further confirmed poor differentiation to be an independent risk factor for OLNM (OR: 1.883, 95% CI: 1.392–31.05, *p* = 0.017). The observed OLNM rates across different histological grades of differentiation were as follows: high differentiation (6, 16.2%), moderate differentiation (4, 19.0%), and low differentiation (10, 58.8%). The WHO recognizes 22 distinct histological subtypes of salivary gland tumors [30]. Given this diversity, the tumors were categorized into two pathological cohorts: low‐grade and non‐low‐grade tumor types. The characteristics and OLNM rates for these cohorts are detailed in Tables 3 and 4. The heterogeneity in histological subtypes and the corresponding biological processes contribute to variable risks of lymph node metastasis [2, 30, 31]. Histologic grading is widely regarded as a critical determinant of lymph node dissemination in PC. Tumor subtypes such as undifferentiated carcinoma, squamous cell carcinoma, salivary duct carcinoma, nonspecific adenocarcinoma, and high‐grade mucoepidermoid carcinoma show OLNM risks exceeding 50%. In contrast, acinar cell carcinoma, secretory carcinoma, adenoid cystic carcinoma, and low‐grade mucoepidermoid carcinoma demonstrate substantially lower OLNM risks, estimated at 2%–4% [21, 32, 33, 34, 35]. Notably, pleomorphic adenoma has been reported to show a lymph node metastasis rate as high as 52% in certain contexts [21]. High‐grade parotid malignancies are associated with a markedly increased likelihood of OLNM compared with low‐grade tumors. In a retrospective analysis of 142 patients with cN0 PC, including 90 who underwent neck dissection, Klussmann et al. [29] reported an OLNM incidence of 49% among high‐grade malignancies. Consistent with these findings, our study (Tables 3 and 4) demonstrated significantly higher OLNM rates in non‐low‐grade tumor subtypes, including ductal carcinoma (80%), adenocarcinoma (75%), lymphoepithelial carcinoma (50%), and squamous cell carcinoma (30%), compared to their low‐grade counterparts.

Additionally, a paucity of clinical trials evaluating the prognostic implications of neck dissection in patients with cN0 poses a significant challenge in establishing evidence‐based guidelines for surgical management. Previous studies have identified histologic grading as a pivotal determinant of prognosis, with poorly differentiated tumors strongly associated with adverse outcomes [36, 37, 38]. In the present study, patients were stratified into two cohorts based on tumor differentiation: high differentiation and low‐to‐moderate differentiation. Prognostic outcomes, including PFS and OS, were analyzed in relation to clinical parameters and OLNM rates for low‐grade and non‐low‐grade tumors, respectively. The results revealed no significant differences in PFS or OS between patients undergoing neck dissection and those managed without neck dissection within the low‐grade tumor and high‐differentiation cohorts (*p* > 0.05). Similarly, for low‐grade tumors with low‐to‐moderate differentiation, no significant differences in OS were observed between the two treatment groups (*p* > 0.05). However, among patients with non‐low‐grade tumors and low‐to‐moderate differentiation, the neck dissection cohort showed significantly improved PFS compared to the no‐neck dissection cohort (*p* < 0.05). In addition, there were also no significant differences between the no‐neck and the neck dissection cohort on OS for overall patients (*p* > 0.05). However, the neck dissection cohort had a better PFS than the no‐neck dissection cohort for overall patients (*p* < 0.05).

While these risk factors are well documented, their application in clinical decision‐making remains challenging. Preoperative determination of definitive histology and tumor grade is frequently unattainable, and high‐risk histological features associated with OLNM often necessitate comprehensive pathological evaluation of resected specimens. Fine‐needle aspiration cytology, despite being a widely utilized diagnostic modality, has limited accuracy in determining precise histological type and grade, with reported diagnostic accuracy rates ranging from 51% to 79% [7, 8]. This accuracy may improve in specialized centers with experienced cytopathologists [39].

Intraoperative frozen section analysis offers valuable insights into malignancy grading [40]. However, its utility is restricted by the inability to facilitate preoperative planning or multidisciplinary team discussions. Recent advances in molecular medicine have identified potential molecular markers associated with lymphatic metastasis in head and neck malignancies, and yet, data on their prognostic utility in salivary gland cancers remain sparse [41, 42]. Additionally, the risk of OLNM in cN0 patients is influenced by the diagnostic techniques used for neck staging. Over recent decades, significant advancements in diagnostic modalities have resulted in substantial differences in the characterization of clinically negative necks compared to historical practices. These advancements limit the applicability of findings from earlier studies to contemporary clinical practice [43].

## 5 Conclusion

The management of cN0 PC remains a complex and nuanced challenge. Treatment strategies should be guided by the presence of established clinical and histopathological risk factors. Notably, patients with poorly differentiated tumor subtypes have a higher propensity for OLNM. For cases of cN0 PC, prophylactic neck dissection may confer a PFS benefit in individuals with higher grade or low‐to‐intermediate differentiation tumors. However, this intervention does not appear to impact OS. Therefore, the selective application of neck dissection should be reserved for these specific patient cohorts to optimize therapeutic outcomes.

## Author Contributions

**Yudong Ning:** conceptualization (equal), data curation (equal), formal analysis (equal), funding acquisition (equal), investigation (equal), methodology (equal), project administration (equal), resources (equal), software (equal), supervision (equal), validation (equal), visualization (equal), writing – original draft (equal), writing – review and editing (equal). **Yixuan Song:** conceptualization (equal), data curation (equal), formal analysis (equal), funding acquisition (equal), investigation (equal), methodology (equal). **Yuqin He:** conceptualization (equal), data curation (equal), formal analysis (equal), funding acquisition (equal), investigation (equal), methodology (equal), project administration (equal). **Han Li:** data curation (equal), formal analysis (equal), funding acquisition (equal). **Shaoyan Liu:** supervision (equal), writing – review and editing (equal).

## Ethics Statement

The study protocol was approved by the Ethics Committee of Cancer Hospital, Chinese Academic of Medical Science (Approval No. NCC‐003094), and it was compliant with the Helsinki Declaration of 1975, as revised in 2008.

## Consent

All patients provided written informed consent at the time of entering this study.

## Conflicts of Interest

The authors declare no conflicts of interest.

## Supporting information

Supporting information.

## Data Availability

Data generated in this study are available upon reasonable request from the corresponding author.
