# Supplementary material for: Prognostic Effect of Neck Dissection and Risk Factors for Occult Lymph Node Metastasis in cN0 Parotid Carcinoma
Source: Cancer Innov. 2025 Apr 7;4(3):e70007. doi: 10.1002/cai2.70007 (PMC11973498; doi:10.1002/cai2.70007)
Supplement: Supplementary file 1 — Supporting information. [file CAI2-4-e70007-s001.doc]

Supplementary Table 1 The baseline between no neck dissection and neck dissection before PSM

| Clinical characters | No neck dissection  *N*=58, *n*(%) | Neck dissection  *N*=75, *n* (%) | *p* value |
| --- | --- | --- | --- |
| Gender |  |  |  |
| Female | 34(58.6) | 38(50.7) | 0.361 |
| Male | 24(41.4) | 37(49.3) |  |
| Age(years old) |  |  |  |
| <60 | 44(75.9) | 50(66.7) | 0.248 |
| ≥60 | 14(24.1) | 25(33.3) |  |
| Tumor Types(low grade malignancy) |  |  |  |
| No | 22(37.9) | 38(50.7) | 0.143 |
| Yes | 36(62.1) | 37(49.3) |  |
| Differentiation |  |  | |
| High | 45(77.6) | 37(49.3) | 0.003 |
| Mid | 5(8.6) | 21（28.0） |  |
| Poor | 8(13.8) | 17（22.7） |  |
| Facial nerve invaved |  |  |  |
| No | 36(63.2) | 40(53.3) | 0.258 |
| Yes | 21(36.8) | 35(46.7) |  |
| Facial nerve reserved |  |  |  |
| No | 5(8.6) | 9(12.0) | 0.529 |
| Yes | 53(91.4) | 66(88.0) |  |
| T State |  |  |  |
| T1 | 30(51.7) | 24(32.0) | 0.097 |
| T2 | 25(43.1) | 44(58.7) |  |
| T3 | 3(5.2) | 5(6.7) |  |
| T4 | 0(0) | 2(1.5) |  |

PSM: Propensity score matching

Supplementary Table 2 The baseline between no neck dissection and neck dissection after PSM

| Clinical characters | No neck dissection  *N*=50, *n*(%) | Neck dissection  *N*=50, *n* (%) | *p* value |
| --- | --- | --- | --- |
| Gender |  |  |  |
| Female | 28(56.0) | 28(56.0) | 1 |
| Male | 22(44.0) | 22(44.0) |  |
| Age(years old) |  |  |  |
| <60 | 37(74.0) | 37(74.0) | 1 |
| ≥60 | 13(26.0) | 13(26.0) |  |
| Tumor Types(low grade malignancy) |  |  |  |
| No | 18(36.0) | 21(42.0) | 0.539 |
| Yes | 32(64.0) | 29(58.0) |  |
| Differentiation |  |  | |
| High | 37(74.0) | 31(62.0) | 0.176 |
| Mid | 5(10.0) | 12（24.0） |  |
| Poor | 8(16.0) | 7（14.0） |  |
| Facial nerve invaved |  |  |  |
| No | 32(64.0) | 31(62.0) | 0.836 |
| Yes | 18(36.0) | 19(38.0) |  |
| Facial nerve reserved |  |  |  |
| No | 4(8.0) | 6(12.0) | 0.505 |
| Yes | 46(92.0) | 44(88.0) |  |
| T State |  |  |  |
| T1 | 25(50.0) | 21(42.0) | 0.673 |
| T2 | 22(44.0) | 25(50.0) |  |
| T3 | 3(6.0) | 3(6.0) |  |
| T4 | 0(0) | 1(2.0) |  |

PSM: Propensity score matching
